# Supplementary material for: A machine learning approach for rapid early detection of Campylobacter spp. using absorbance spectra collected from enrichment cultures
Source: PLoS One. 2024 Sep 6;19(9):e0307572. doi: 10.1371/journal.pone.0307572 (PMC11379395; doi:10.1371/journal.pone.0307572)
Supplement: S1 File — (DOCX) [file pone.0307572.s001.docx]

# **A novel approach for *Campylobacter* spp. detection: spectrophotometry helps with early prediction**

Kefeng Zhang, Christelle Schang, Rebekah Henry, David McCarthy

# **Supporting information – S1 File**

**S1 Table**. Summary of parameter ranges in sensitivity test and the final selected values

| **Parameters^*^** | **Range** | **Chosen parameter value after test** | | |
| --- | --- | --- | --- | --- |
|  |  | **Even controls** | **Controls** | **Water samples only** |
| **Logistic regression** | | | | |
| C | 0.0001 to 1 | 1.0 | 1.0 | 0.1 |
| penalty | *l1*, *l2*, *elasticnet*, *none* | l2 | l2 | l2 |
| Tol | 1e-5 to 0.1 | 1e-5 | 1e-5 | 1e-5 |
| Solver | *newton-cg*, *lbfgs*, *liblinear*, *saga* | lbfgs | lbfgs | Lbfgs |
| class_weight | 1:1, 1:2, 1:5, 1:10, 1:20, 1:30, 1:50, 1:100, 1:200, 1:500, 1:1000, ‘balanced’ | ‘balanced’ | ‘balanced’ | ‘balanced’ |
| **SVM** | | | | |
| C | 0.001 to 1 | 0.03 | 0.03 | 0.1 |
| Kernel | *linear*, *poly*, *rbf*, *sigmoid* | linear | linear | linear |
| gamma | *scale*, *auto* | Scale | Scale | Scale |
| Tol | 1e-5 to 0.1 | 1e-5 | 1e-5 | 1e-5 |
| Class_weight | 1:1, 1:2, 1:5, 1:10, 1:20, 1:30, 1:50, 1:100, 1:200, 1:500, 1:1000, ‘*balanced’* | ‘balanced’ | ‘balanced’ | ‘balanced’ |
| **Random forest** | | | | |
| N_estimate | 10-1000 | 100 | 100 | 100 |
| Max_depth | 2-30 | 7 | 7 | 10 |
| Criterion | *gini*, *entropy* | *gini* | *gini* | *gini* |
| Max_feature | *auto*, *sqrt*, *log2* | *auto* | *auto* | *auto* |
| boostrap | Ture, False | Ture | Ture | Ture |

Note * Refer to the link for explanation of all parameters for each machine learning approach:

- Logistic regression: <https://scikit-learn.org/stable/modules/generated/sklearn.linear_model.LogisticRegression.html>
- SVM: <https://scikit-learn.org/stable/modules/generated/sklearn.svm.SVC.html>
- Random forest: <https://scikit-learn.org/stable/modules/generated/sklearn.ensemble.RandomForestClassifier.html>

It shall be noted that more parameters could be added into each ML approach, however the focus of this study was not to have comprehensive understanding of how these will impact the results, thus the authors decided to select these ones in the table that are believed to be more important.

**S2 Table.** Number of samples that have two distinctive peaks at 540-542nm and 575-576nm

| **Site** | **Pos samples** | **Neg samples** |
| --- | --- | --- |
| Controls | 58/63 = 92.1% | 11/142 = 7.8% |
| All | 496/525 = 94.5% | 445/1050 = 42.4% |
| Rural catchment | 38/38 =100 % | 143/226 = 62.3% |
| Urban catchment | 92/96 = 95.9% | 23/91 = 25.3% |
| Mixed rural and urban catchment | 358/391 = 91.6% | 242/699 = 35.1% |

**S3 Table.** Summary of the model performance (based on training) under different test scenarios.

| **Test scenario** | | **Accuracy** | | | **False negative rate (FNR)** | | |
| --- | --- | --- | --- | --- | --- | --- | --- |
|  |  | **SVM** | **Logistic** | **RF** | **SVM** | **Logistic** | **RF** |
| 1 | *C_all_ + W_all_* | 0.726 | 0.720 | 0.744 | 10.5% | 10.3% | 15.5% |
| 2 | *C_even_ + W_all_* | 0.969 | 0.969 | 1.000 | 4.2% | 4.2% | 0.0% |
| 3 | *W_all,80_ + W_all,20_* | 0.745  (0.011) | 0.775  (0.008) | 0.893  (0.007) | 12.4%  (0.7%) | 13.0%  (0.4%) | 6.6%  (0.7%) |
| 4 | *C_all_ + W_Rural_* | 0.985 | 0.980 | 1.000 | 3.2% | 3.2% | 0.0% |
| 5 | *W_Rural,80_ + W_Rural,20_* | 0.487  (0.028) | 0.635  (0.034) | 0.998  (0.002) | 14.4%  (3.8%) | 19.5%  (4.0%) | 0.0%  (0.0%) |
| 6 | *C_all_* + *W_Urban_* | 0.985 | 0.980 | 1.000 | 3.2% | 3.2% | 0.0% |
| 7 | *W_Urban,80_ + W_Urban,20_* | 0.868  (0.010) | 0.896  (0.010) | 0.994  (0.009) | 3.5%  (0.5%) | 4.0%  (1.0%) | 0.0%  (0.0%) |
| 8 | *C_all_* + *W_Mix_* | 0.985 | 0.980 | 1.000 | 3.2% | 3.2% | 0.0% |
| 9 | *W_Mix,80_ + W_Mix,20_* | 0.746  (0.002) | 0.787%  (0.003) | 0.909  (0.007) | 14.0%  (0.7%) | 14.6%  (0.7%) | 5.8%  (1.3%) |

|  |  |  |  |  |  |
| --- | --- | --- | --- | --- | --- |
