# Supplementary material for: A machine learning approach for rapid early detection of Campylobacter spp. using absorbance spectra collected from enrichment cultures
Source: PLoS One. 2024 Sep 6;19(9):e0307572. doi: 10.1371/journal.pone.0307572 (PMC11379395; doi:10.1371/journal.pone.0307572)
Supplement: Attachment — Submitted filename: Zhang et al_R1 Response to Reviewers.docx [file pone.0307572.s003.docx]

# Response to Reviewers

## **Referee: 1**

|  | Comment | Response |
| --- | --- | --- |
| **General comments** | | |
|  | Reviewer #1: Dear authors,  thank you for submitting your research, it is very interesting and relevant work you do!  Overall, I like your submission. You explore the potential of ML methods improving the speed and potentially the quality of the assessment of probes for the presence of Campylobacter.  The introduction to the topic is clear and concise, so is the description of the employed data collection and ML methods.  As my expertise lies in the ML area, I will focus my comments on the corresponding parts of the submission. To summarize, I have major concerns with the training, testing and evaluation procedures of the ML methods, and a list of minor comments for improving the manuscript. | Thank you for your positive feedback and for recognizing our research's relevance and potential impact on improving the assessment of probes for the presence of Campylobacter using ML methods. We appreciate your compliments on the clarity and conciseness of our introduction and methodology descriptions.  We did some serious tests of the models regarding the selection of hyperparameters and the full model tests. We have clarified this in the revised manuscript and responded to your specific comments in this revision note below.  We hope our manuscript has been improved and you are happy with the revision. |
|  | It is good to start with simple methods like logistic regression, SVM and RF. However, the evaluation should be thorough. Most importantly, it was not entirely clear how the training and hyperparameter selection was performed. To select the hyperparameters, one should perform a N-fold cross validation for each hyperparameter, e.g., split the training data into 80-20 random splits and train and test on different splits for each hyperparameter, then select the one with the lowest average test performance.  After this selection process, the method should be trained on the entire training data set again using the selected hyperparameters and then finally applied to the actual test set which was not used at all before.  It is essential the report error bars on the report classifier performances (Table 3). To that end, one could repeat the above procedure 5-10 (N) times and report mean test performance +- standard error of the mean (mean +- std / sqrt(N).  What is the dimensionality of the spectral data? If it is high, this might be an explanation for the relatively low performance of the classifiers. It might be worth exploring non-linear methods, e.g., neural-network based logistic regression. However, I see that the training data is limited and might not suffices for training neural networks. | Thank you for your comment.  We agree that the evaluation needs to be very thorough, even with simple methods.  In Section 2.3.2 Preliminary testing, we introduced how the hyperparameters were selected. We first ran the model 1000 times (with random 80-20 data split for training and testing) to gauge the range and sensitivity of the hyperparameters that were thought to impact the model performance. Thereafter, we ran another 1,000 runs to determine the impact of hyperparameters with a refined range of values. We found that most parameters were insensitive, and thus, default values were set.  We have changed some sentences for better clarity. Supporting Information S1 Table also gives the initial ranges of the hyperparameters tested and the final selected hyperparameter values.  For Scenarios 1, 2, 4, 6, and 8 in Table 3, the training and testing datasets were always the same (as indicated in Table 2). For training, the control samples were used, while for testing, the water samples from specific locations (or all locations) were used. Thus, although we repeated the model testing five times, the results were almost identical.  However, for scenarios 3,5, 7, and 9, the 80-20 split for training and testing was different every time, and thus, the five repeated runs led to different results. In Table 3, we now have included the average accuracy and false negative rate, with their standard deviations from five repeated runs. The corresponding results and discussions have been updated.  We have also included the training results in the Supporting Information S3 Table.  Spectral data has wavelengths from 453 nm to 800 nm. In preliminary testing, we tried to take a subset of wavelengths of spectral data based on feature selection. Still, the performance was only marginally lower than when using the full spectrum. Thus, we have decided to use all, as this would be more applicable to future use. Testing NN-based logistic regressions could be a good option moving forward, but we hope to do this in the future studies after collecting more data. |
| Further questions and questions: | | |
| 1 | 1) How exactly is the MPN calculated? In line 170 you mention “by applying probability theory”. I think this part definitively need more explanation, given that it seems to be the central assessment for the presence of Campylobacter. I am also wondering what it is needed at all. Why not report the presence of the bacteria as binary variable and take the average over probes? | Thank you for highlighting the need for a clearer explanation of the Most Probable Number (MPN) calculation in our manuscript.  The method for calculating MPN is well developed in the literature, and we used a very standard method, with the most relevant literature cited, e.g., Briones and Reichardt (1999); Garthright and Blodgett (2003), and (McBride et al., 2003). We also added more explanations of this MPN calculation method in Section 2.2.3, including the following added sentences (Lines 176 – 191 of the manuscript with tracked changes):  *“This theory calculates the probability that a particular tube among replicates will contain at least one bacterium (in this case, Campylobacter), indicated by a positive response after incubation. We can determine the probability of each pattern by considering all possible combinations over a range of bacterial numbers (n). From the resulting bar graph of these probabilities versus n, we can identify the Most Probable Number (MPN) - the value of n for the highest bar divided by the total volume in the test setup - and its occurrence probability(McBride et al., 2003).”*  We believe that reporting MPN is important, the rationale for using MPN over a binary presence/absence reporting method includes:   - Quantitative Assessment: MPN provides a quantitative estimate of bacterial concentration, which is more informative for assessing contamination levels and potential health risks. - Probability Theory: By applying probability theory, MPN can give a statistically robust estimate even when the bacterial concentrations are low, leveraging the entire pattern of observed growth rather than a simple average of binary outcomes. - Sensitivity and Specificity: MPN can be more sensitive and specific in detecting low concentrations of bacteria than binary methods, which might oversimplify the assessment and miss subtle variations in bacterial presence. |
| 2 | How exactly is the correlation between spectral data (continuous, high-dimensional) and Campylobacter presence (binary) calculated? | Thanks for your comment.  We performed a simple Pearson correlation analysis to estimate the linear relationships between the absorbance data (full spectrum from 453 nm to 850 nm) and the presence/absence of Campylobacter based on the PCR analysis results.  In section 2.3.1, we tried to clarify this method (Lines 205 – 207):  *“Pearson correlation analysis was performed by using IBM SPSS Statistics software to understand the linear relationships between the absorbance data (full spectrum from 453 nm to 850 nm) and the presence/absence of Campylobacter based on PCR analysis results.”*  Supporting information S2 File Correlation results was included to show the correlation results. |
| 3 | RF should also use the class_weight="balanced" option because the number of positive and negative examples is not equal. | Thanks for the comment, yes, we used “balanced” option – refer to SI for more details, and we did preliminary experiment. |
| 4 | Table 3: the extraordinary high training accuracy is a strong sign for overfitting! Also, how can the test accuracy be 1.0 for test 5? This looks suspicious. Error bars would help here (see comment above). | Thank you for the comments.  We checked the results of this scenario 5 and found some mistakes, which are now fixed. We have now put the average performance (with standard deviation) of ACC and FNR in Table 3. |
| 5 | Figure 5: Absolute numbers are difficult to interpret. Numbers should be relative, e.g., percentage or proportions. | Thanks for your comment.  We usually do not report NSE values in percentage or proportions. Please refer to the response to your next comment, in which we clarified why we used NSE, how it was calculated, and what NSE values indicate. |
| 6 | The justification for using NSE for comparing the MPN predictions is not clear. Also, the explanations for how exactly NSE and the confidence intervals are calculated are missing. As a consequence, Figures 5 and 6 are unclear, e.g., why can NSE be negative and what does that mean? | Thanks for your comment. We have added a lot more details about the use of NSE, how it was calculated, and what the NSE values tell us in Section 2.2.3 (Lines 284-296):  *“Nash-Sutcliffe efficiency (NSE) coefficient (Nash and Sutcliffe, 1970), which is widely used for the assessment of water quality models (e.g., (Bosco et al., 2023; Kalin et al., 2003; Zhang et al., 2019a)), was used in this study to evaluate the ability of MPN-Spectro-ML method in predicting MPN. The NSE is calculated using the following formula:*  $NSE=1-\frac{\sum_{i=1}^{n} {(O_{i}-P_{i})}^{2}}{\sum_{i=1}^{n} {(O_{i}-\bar{O})}^{2}}$  *Oi is the MPN values estimated from the MPN-PCR method (considered as the observed value); Pi is the MPN values estimated from the MPN-Spectro-ML method (considered as the predicted value).* $\bar{O}$ *is the mean of the observed values (i.e., all MPN values from the MPN-PCR method). The NSE ranges from* $-\infty$ *to 1, with 1 indicating a perfect match between observed and predicted values. When NSE equals zero, the predictive power is equivalent to simply using the average of the observed values as the prediction for all time steps, while negative NSE values indicate that the model predictions are worse than using the mean of the observed data. Zhang et al. (2019b) suggested that NSE values greater than 0.3 indicate moderate model performance, while NSE values less than 0.3 indicate poor model performance.”*  As for the confidence intervals of MPN values, it is part of MPN estimation process, and we used Haldane’s approximation (Haldane, 1939) to estimate the 95% confidence intervals. This is indicated in section 2.2.3 (Lines 190-191). |
|  | Minor comments | Thank you for all the detailed feedback. We have addressed them below. |
|  | line 15: first sentence seems grammatically off and difficult to parse | We have revised it into (Lines 17-18):  “Enumeration of Campylobacter from environmental waters can be challenging due to its low concentrations, which can still pose a significant health risk.” |
|  | line 39: “-a” | Removed “-” |
|  | line 49: change of font size. | Font size has been made consistent across the main parts of the manuscript. |
|  | line 58: MPN-PCR has not been introduced | Revised into:  “Most-Probable Number (MPN)-PCR” |
|  | line 60: AS/NZS not introduced | Changed into:  “the Australian/New Zealand Standard (AS/NZS)” |
|  | line 85: acronyms not introduced | Full names are now given, and we also double-checked across the manuscript for the use of those acronyms. |
|  | line 89: Argument is not sound. How does this sentence follow from the previous sentence? | We have revised the sentence (Lines 94-96):  “Consequently, the potential of using spectrophotometry coupled with machine learning models to predict the presence of pathogens (e.g., Campylobacter) in these complex matrices remains unexplored.” |
|  | line 107: Typo “these”, “spp” | Fixed. |
|  | line 199: Unlikely that this is the correct reference for logisitic regression or the sigmoid function. | Thanks for your comment. We have revised this sentence with updated references (Lines 216 – 223):  “The first ML method used was logistic regression (or logit regression, LR), a statistical model that has been used for water quality simulations (e.g., (Nallakaruppan et al., 2024)). In this study, we used LR to find the probability of Campylobacteria presence (p) in the collected water samples. It learns a linear relationship between independent variables (i.e., in this case, absorbance at different wavelengths) and the log-odds (the ratios of the probabilities of the event happening to it not happening, i.e., log(p/(1-p))) from the given dataset (Nallakaruppan et al., 2024; Peng et al., 2002).” |
|  | line 420: what are “good” correlations? I suggest to use a different more objective adjective. | Thanks for your comment; we have revised this sentence into (Lines 478-480):  *“This study shows significant correlations (r > 0.40, p<0.01) between Campylobacter presence/levels and the absorbance of 56 wavelengths in the range of 531 nm to 586 nm)”* |

**Journal requirements**

|  | **Comment** | **Response** |
| --- | --- | --- |
| 1 | Please ensure that your manuscript meets PLOS ONE's style requirements, including those for file naming. The PLOS ONE style templates can be found at   - <https://journals.plos.org/plosone/s/file?id=wjVg/PLOSOne_formatting_sample_main_body.pdf> - https://journals.plos.org/plosone/s/file?id=ba62/PLOSOne_formatting_sample_title_authors_affiliations.pdf | We have made changes to meet the journal requirements, this includes formatting:   - heading/subheading, - figures, - tables, - equation, - supporting document references, - references, - title page, |
| 2 | In your Methods section, please provide additional information regarding the permits you obtained for the work. Please ensure you have included the full name of the authority that approved the field site access and, if no permits were required, a brief statement explaining why. | We did have all the permits for the fieldwork. A sentence has been added to the method:  *“The permits for accessing all the sites were acquired from the asset owner Melbourne Water.”* |
|  | 3. Thank you for stating the following financial disclosure:  “The first and corresponding author (Kefeng Zhang) is supported by Australian Research Council Discovery Early Career Researcher Award (ARC DECRA, DE210101155). The data collected and used in this paper were collected as part of two different Australian Research Council Linkage Projects (LP120100718, LP160100408) and another ARC DECRA (DE140100524). The authors would like to acknowledge Melbourne Water and EPA Victoria for co-funding of the ARC LPs.”  Please state what role the funders took in the study. If the funders had no role, please state: "The funders had no role in study design, data collection and analysis, decision to publish, or preparation of the manuscript."  If this statement is not correct you must amend it as needed.  Please include this amended Role of Funder statement in your cover letter; we will change the online submission form on your behalf. | This is indicated in the cover letter:  *“The funders have provided permits to the sites for data collection. They also provided some constructive feedback on the manuscript and acknowledged the submission of this manuscript.”* |
|  | 4. Thank you for stating the following in the Acknowledgments Section of your manuscript:  “The first and corresponding author (Kefeng Zhang) is supported by Australian Research Council Discovery Early Career Researcher Award (ARC DECRA, DE210101155). The data collected and used in this paper were collected as part of two different Australian Research Council Linkage Projects (LP120100718, LP160100408) and another ARC DECRA (DE140100524). The authors would like to acknowledge Melbourne Water and EPA Victoria for co-funding of the ARC LPs. Dr Rhys Coleman, Dr Nick Crosbie, and Dr Melita Stevens are also greatly acknowledged for providing constructive feedback to the manuscript.”  We note that you have provided funding information that is currently declared in your Funding Statement. However, funding information should not appear in the Acknowledgments section or other areas of your manuscript. We will only publish funding information present in the Funding Statement section of the online submission form.  Please remove any funding-related text from the manuscript and let us know how you would like to update your Funding Statement. Currently, your Funding Statement reads as follows:  “The first and corresponding author (Kefeng Zhang) is supported by Australian Research Council Discovery Early Career Researcher Award (ARC DECRA, DE210101155). The data collected and used in this paper were collected as part of two different Australian Research Council Linkage Projects (LP120100718, LP160100408) and another ARC DECRA (DE140100524). The authors would like to acknowledge Melbourne Water and EPA Victoria for co-funding of the ARC LPs.”  Please include your amended statements within your cover letter; we will change the online submission form on your behalf. | We are happy to keep the following text as the funding information:  “The first and corresponding author (Kefeng Zhang) is supported by Australian Research Council Discovery Early Career Researcher Award (ARC DECRA, DE210101155). The data collected and used in this paper were collected as part of two different Australian Research Council Linkage Projects (LP120100718, LP160100408) and another ARC DECRA (DE140100524). The authors would like to acknowledge Melbourne Water and EPA Victoria for co-funding of the ARC LPs.”  This has been removed from the acknowledgment section. |
|  | 5. PLOS requires an ORCID iD for the corresponding author in Editorial Manager on papers submitted after December 6th, 2016. Please ensure that you have an ORCID iD and that it is validated in Editorial Manager. To do this, go to ‘Update my Information’ (in the upper left-hand corner of the main menu), and click on the Fetch/Validate link next to the ORCID field. This will take you to the ORCID site and allow you to create a new iD or authenticate a pre-existing iD in Editorial Manager. Please see the following video for instructions on linking an ORCID iD to your Editorial Manager account: https://www.youtube.com/watch?v=_xcclfuvtxQ. | Done as suggested |
|  | 6. Please include captions for your Supporting Information files at the end of your manuscript, and update any in-text citations to match accordingly. Please see our Supporting Information guidelines for more information: http://journals.plos.org/plosone/s/supporting-information. | We have included captions at end of the manuscript for supporting information. |

Bosco, C., Abdalla, E.M.H., Muthanna, T.M., Alfredsen, K., Rasten, B., Kjennbakken, H. and Sivertsen, E. 2023. Evaluating the Stormwater Management Model for hydrological simulation of infiltration swales in cold climates. Blue-Green Systems 5(2), 306-320.

Briones, A.M. and Reichardt, W. 1999. Estimating microbial population counts by ‘most probable number’ using Microsoft Excel®. Journal of Microbiological Methods 35(2), 157-161.

Garthright, W.E. and Blodgett, R.J. 2003. FDA's preferred MPN methods for standard, large or unusual tests, with a spreadsheet. Food Microbiology 20(4), 439-445.

Haldane, J.B. 1939. Sampling errors in the determination of bacterial or virus density by the dilution method. J Hyg (Lond) 39(3), 289-293.

Kalin, L., Govindaraju, R.S. and Hantush, M.M. 2003. Effect of geomorphologic resolution on modeling of runoff hydrograph and sedimentograph over small watersheds. Journal of Hydrology 276(1), 89-111.

McBride, G.B., McWhirter, J.L. and Dalgety, M.H. 2003. Uncertainty in most probable number calculations for microbiological assays. J Aoac Int 86(5), 1084-1088.

Nallakaruppan, M.K., Gangadevi, E., Shri, M.L., Balusamy, B., Bhattacharya, S. and Selvarajan, S. 2024. Reliable water quality prediction and parametric analysis using explainable AI models. Scientific Reports 14(1), 7520.

Peng, C.-Y.J., Lee, K.L. and Ingersoll, G.M. 2002. An Introduction to Logistic Regression Analysis and Reporting. The Journal of Educational Research 96(1), 3-14.

Zhang, K., Deletic, A., Bach, P.M., Shi, B., Hathaway, J.M. and McCarthy, D.T. 2019a. Testing of new stormwater pollution build-up algorithms informed by a genetic programming approach. Journal of Environmental Management 241, 12-21.

Zhang, K., Randelovic, A., Deletic, A., Page, D. and McCarthy, D.T. 2019b. Can we use a simple modelling tool to validate stormwater biofilters for herbicides treatment? Urban Water Journal 16(6), 412-420.
